# Effect of Testosterone Treatment on Cardiovascular Events in Men: Protocol for a Systematic Literature Review and Meta-Analysis

**DOI:** 10.2196/15163

**Published:** 2020-10-29

**Authors:** HuiJun Chih, Christopher M Reid, Bu B Yeap, Girish Dwivedi

**Affiliations:** 1 School of Public Health Curtin University Perth Australia; 2 School of Public Health & Preventative Medicine Monash University Melbourne Australia; 3 Medical School University of Western Australia Perth Australia; 4 Department of Endocrinology and Diabetes Fiona Stanley Hospital Perth Australia; 5 Harry Perkins Institute of Medical Research University of Western Australia Perth Australia; 6 Department of Cardiology Fiona Stanley Hospital Perth Australia

**Keywords:** exogenous testosterone, stroke, nonfatal myocardial infarction, emergency revascularization, carotid surgery, cardiac mortality

## Abstract

**Background:**

Testosterone prescriptions have increased dramatically in recent decades, with increasing usage in men. Despite epidemiological associations reported high circulating concentrations of endogenous androgens and low risk of cardiovascular events and mortality, the effects of exogenous androgens in the form of testosterone therapy for maintaining physiological circulating androgen concentrations on the cardiovascular system remain uncertain with no published meta-analysis on this topic.

**Objective:**

The aim of this study was to investigate the effects of prescribed testosterone treatment, in all forms and durations, from well-developed randomized controlled trials, on cardiovascular events in men aged 18 years or older.

**Methods:**

Peer-reviewed journal articles published from 1980 to 2019 will be searched from databases (ie CINAHL [Cumulated Index to Nursing and Allied Health Literature], Embase, Medline, Scopus, Cochrane Controlled Register of Trials as well as the Clinical Trial Registry). Randomized controlled trials or cluster randomized controlled trials with at least one intervention arm of testosterone and a control group of usual care or no testosterone treatment will be included in this review and meta-analysis. Studies on men with previous cardiovascular events or cardiac vascularization (coronary bypass surgery or percutaneous coronary intervention) will be excluded. Data related to primary outcomes such as clinical events of any type of stroke or transient ischemic attack, nonfatal myocardial infarction or acute coronary syndrome, emergency coronary artery revascularization, carotid surgery, cardiac mortality, and all-cause mortality will be extracted for analysis. The criteria for PRISMA (Preferred Reporting Items for Systematic Reviews and Meta-Analyses) will be followed in the evaluation of evidence.

**Results:**

Search terms have been piloted and finalized. This study will be completed by the end of 2020.

**Conclusions:**

This protocol will guide a systematic literature review of the evidence around prescribed testosterone and its effect on cardiovascular events in men aged 18 years or older. The findings will inform clinical management of hypogonadal men.

**Trial Registration:**

PROSPERO International Prospective Register of Systematic Reviews CRD42019134278; https://tinyurl.com/y6t7ggge

**International Registered Report Identifier (IRRID):**

PRR1-10.2196/15163

## Introduction

Epidemiological observational studies have shown that low endogenous testosterone concentrations are associated with a high incidence of cardiovascular events and cardiovascular mortality in middle-aged and older men [1-3]. Potential mechanisms by which testosterone could exert beneficial effects on the vasculature include reduction in cholesterol accumulation, modulation of inflammation, and improvement in endothelial function [4,5]. However, this causation is unproven as there has been no randomized placebo-controlled trial of testosterone sufficiently powered to examine the outcomes of cardiovascular events or mortality.

Small randomized trials of testosterone therapy have shown improvements in surrogate endpoints related to cardiovascular risk [6-9]. However, other trials of testosterone have not shown improvements in carotid atherosclerosis, which was assessed using carotid intima-media thickness. One study reported an increase in noncalcified coronary atheroma, which was assessed by coronary computed tomography angiography in older men receiving testosterone therapy over a period of 12 months [10]. While 1 randomized trial of testosterone therapy in older men with mobility limitations reported an excess of adverse events in the treatment arm [11], a recent large trial in older men did not find any excess of cardiovascular adverse events with testosterone treatment during the intervention period [12]. Indeed, the effects of exogenous androgens in the form of testosterone therapy for maintaining the physiological circulating androgen concentrations on the cardiovascular system remain uncertain.

To date, there is no meta-analysis published on this topic that focuses on the outcomes of randomized controlled trials. It is therefore challenging for clinicians to decide if any testosterone should be prescribed to hypogonadal men. Owing to this evidence gap for the current clinical management of hypogonadal men, a systematic literature review and meta-analysis of data from well-designed randomized controlled trials is needed to ascertain the beneficial, neutral, or adverse effects of testosterone treatment on cardiovascular outcomes in the general population of middle-aged and older men. The aim of this review is to systematically assess the evidence of the effects of testosterone treatment (either in the form of oral administration, transdermal application, intramuscular, or implant) in comparison to those of no testosterone treatment on cardiovascular outcomes of men aged 18 years or older.

## Methods

### Review Objectives and Hypotheses

This systematic review and meta-analysis will follow the PRISMA-P (Preferred Reporting Items for Systematic Review and Meta-Analysis Protocols) [13] for evaluating the effect of testosterone treatment on primary cardiovascular outcomes (clinical events of stroke or transient ischemic attack, nonfatal myocardial infarction, or acute coronary syndrome, emergency coronary artery revascularization, carotid surgery, cardiac mortality, and all-cause mortality) among adult men. Three a priori hypotheses will be tested: types of testosterone treatment (oral administration, transdermal application, intramuscular injection, or implant vs no testosterone), dosage of testosterone received (total dosage [mg] received during the duration of the study vs no testosterone), and follow-up period (at least 6 months vs more than 6 months).

### Inclusion Criteria

Studies need to meet the following characteristics to be eligible for this systematic review and meta-analysis.

#### Participants

Studies that examine men aged 18 years or older and report baseline androgen levels will be included. The participants can be overweight or obese, with or without metabolic syndrome, smokers, and of different socioeconomic status. Studies on men with previous cardiovascular events or cardiac vascularization (coronary bypass surgery or percutaneous coronary intervention) will be excluded. Studies that examine both adult men and women will only have the male population data extracted for this review and meta-analysis.

#### Intervention

Testosterone formulation given to humans via oral administration, transdermal application, intramuscular injection, or implant of any dosage and frequency will be examined in this review and meta-analysis.

#### Comparison

The effect of testosterone treatment via oral administration, transdermal application, intramuscular injection, or implant will be compared to that in a control group of usual care or no testosterone intervention.

#### Outcomes

Studies with at least 6-month postintervention follow-up outcomes will be included. The primary postintervention outcomes are clinical events of any type of stroke or transient ischemic attack, nonfatal myocardial infarction or acute coronary syndrome, emergency coronary artery revascularization, carotid surgery, cardiac mortality, and all-cause mortality. Emergency coronary revascularization includes percutaneous coronary intervention or coronary artery bypass graft. The secondary outcome will be any atherosclerotic cardiovascular disease, broadly defined to include events occurring in the coronary, cerebral, aortic, and peripheral vasculature. Therefore, the secondary outcomes will also include ischemic heart disease, coronary heart disease, coronary artery disease, cerebrovascular diseases, including carotid disease, and peripheral vascular disease, including aortic aneurysm. The number of events among those who received any testosterone intervention will be compared to that in the control group of usual care or no testosterone intervention.

### Types of Studies

Randomized controlled trials or cluster randomized controlled trials with at least one intervention arm of testosterone and a control group of usual care or no testosterone treatment will be included. Experimental studies with no randomization (such as quasi-experimental studies) will not be included in this study. Observational cohort studies, case-control studies, nested case-control studies, cross-sectional studies, case series, and case reports will also not be included.

### Search Strategy

Peer-reviewed information published in electronic databases will be searched. Search terms will include testosterone AND (cardiac OR heart OR stroke OR myocardial infarction OR atherosclerosis OR revascularization OR Agatston score OR death OR angina OR carotid OR artery OR coronary OR cerebral OR aortic OR peripheral vascular) anywhere in the text by using truncation and wildcards when available, to accommodate for different spellings. The search modes will be set to Boolean/Phrase when available. In addition, the limiters will be human studies, studies done on males, publications written in English, and publication year set to range from 1980 to 2019 and when available, and peer-reviewed randomized controlled trials. If full-text is not available, the original study authors will be contacted for the full-text of the article published in English language.

### Information Sources

CINAHL (Cumulated Index to Nursing and Allied Health Literature), Embase, Medline, Scopus, and Cochrane Controlled Register of Trials will be searched using the advanced search function, where available. The following search strategy will also be applied when searching the Clinical Trial Registry (ClinicalTrials.gov) for trials with completed results. References of the selected articles will also be scanned to ensure literature saturation.

### Study Selection

The number of records found from each database and the Clinical Trial Registry will be noted in the format of the PRISMA flow diagram. The full details of the articles will be imported to an EndNote library. Duplicates of identical records will be removed and the number of duplicates removed will be recorded in the PRISMA flow diagram. Two independent reviewers will screen the titles and abstracts of the remaining articles. Articles that are conference abstracts, review articles, observational studies, response or letters to the Editors, not on men, on isolated cells or artery specimens, and irrelevant outcomes will be excluded and the number recorded on the PRISMA flow diagram. If consensus is not reached at the screening phase, there will be discussion between the 2 reviewers or a third independent reviewer may be invited to screen the articles until consensus is reached. The remaining full-text articles will be screened against the selection criteria. Full-text articles that do not meet the eligibility criteria will be excluded and the number recorded on the PRISMA flow diagram. If multiple articles were produced from the same active intervention by the same group of authors at the same institution on the same outcome, only the article with the most complete follow-up data will be included in the review. Discussion between 2 independent reviewers or involvement of a third independent reviewer will resolve disagreement on the selection of the articles. References of the selected articles will also be screened as above to ensure literature saturation. The final number of the selected articles will be noted on the PRISMA flow diagram.

### Assessment of the Methodological Quality

The quality of the studies will be assessed by 2 independent reviewers following the Cochrane Collaboration’s tool for assessing risk of bias. The risk of bias will be assessed in terms of sequence generation, allocation concealment, blinding, incomplete outcome data, selective outcome reporting, and bias that may be threats to the validity [14]. Each of these will be assessed as “low risk,” “high risk,” or “uncertain risk” of bias [14] at the study and outcome level. Discussion between 2 independent reviewers and involvement of a third independent reviewer will be required until consensus is reached.

### Data Extraction

The number of articles retrieved and excluded will be recorded in the format of the PRISMA flow diagram. The characteristics of the included study and participants will be noted in a table, grouped by the types of testosterone therapy received. Data extraction will be piloted from a small group of studies by 2 reviewers. One reviewer will then independently extract the data from all the selected articles, which will be verified by the second reviewer. There is no plan of individual patient data meta-analysis; therefore, no further data will be sought from the original researchers. Depending on the number of selected articles, the mean or risk ratios of the outcomes will be presented in a number of tables. Data to be extracted for the review and meta-analysis will include the following: (1) general information of the article (authors, year of publication, country of origin, source of funding), (2) study characteristics (aims of the study, study design, duration of the study, recruitment criteria, sampling technique, unit of randomization [participant or general practitioner]), (3) baseline participant characteristics by intervention group and control group (sample size, age, ethnicity, comorbidities, testosterone level, plaque volume, body mass index or weight classification, smoking status), (4) intervention (generic and trade name of the testosterone therapy, dosage, frequency of administration, and duration of intervention), (5) comparator (usual care or type of placebo for the control group), and (6) outcomes (number lost to follow-up, number of cardiovascular events, odds ratio, risk ratio, hazard ratio, mean [SD] change in volume/score, and effect size). If the data of the key outcomes are not available, the authors of the selected articles will be contacted for the required information.

### Data Synthesis

The quality of the evidence will be assessed against Cochrane’s domains of risk of bias [15]. Meta-biases due to study design or methodological biases, reporting, and publication bias will be assessed. Publication bias will be assessed using Egger’s test and funnel plots [16]. Heterogeneity across studies will be assessed through visual inspection of the forest plot, I^2^ statistic, and τ^2^ [13]. A fixed-effect meta-analysis using the inverse-variance method will be performed if there is minimal heterogeneity [17]. A random-effect model following the DerSimonian and Laird method may be performed to account for heterogeneity (I^2^ statistic >50%) across studies [18]. Possible small sample bias will be assessed by comparing the fixed effect estimate against that from the random effects model. The primary outcomes are the presence of clinical events of any type of stroke or transient ischemic attack, nonfatal myocardial infarction or acute coronary syndrome, emergency coronary artery revascularization, carotid surgery, cardiac mortality, and all-cause mortality. The secondary outcome is any atherosclerotic cardiovascular disease, which includes ischemic heart disease, coronary heart disease, coronary artery disease, cerebrovascular and carotid diseases, aortic aneurysm, and peripheral arterial disease. The number of events regarding primary and secondary outcomes among those who received any testosterone intervention will be compared to that of the control group of usual care or no testosterone intervention and the likelihood of events presented as risk ratios with 95% confidence intervals. Findings regarding coronary atherosclerosis will be reported as changes in the volume of calcified plaque and noncalcified coronary atheromatous plaque or coronary calcium score or Agatston score between baseline and endpoints of the studies (mean [SD]). Carotid atherosclerosis will be reported as the change in the carotid intima-media thickness and volume of carotid plaque between baseline and endpoint of the studies (mean [SD]). The presence of aortic or carotid inflammation on positron emission tomography, in absence of vasculitis, will be reported as the evidence of aortic or carotid atherosclerosis.

Three a priori hypotheses will be tested using stratified meta-analyses. These hypotheses are types of testosterone treatments (oral administration, transdermal application, intramuscular injection or implant vs no testosterone), dosage of testosterone received (total dosage [mg] received during the duration of the study vs no testosterone), and follow-up period (at least 6 months vs more than 6 months). Sensitivity analyses will be performed by excluding studies with high risk of bias and those with pharmaceutical funders. High-risk bias includes any bias rising from issues with allocation concealment (selection bias), blinding (performance bias), incomplete outcome data (attrition bias), selective outcome reporting (reporting bias) [14], or a combination of these issues. The strength of evidence on the effect estimate will be reported as “strong confidence” (no further research will affect the confidence of the effect estimate reported in the current findings), “moderate confidence” (further research may affect the confidence of the effect estimate reported in the current findings), or “uncertain” (further research will most definitely affect the confidence of the effect estimate reported in the current findings).

### Ethics and Dissemination

All data extracted will be in aggregated form with no access to identifiable individual data. No ethics approval is required. This protocol has been registered on PROSPERO (Prospective Register of Systematic Reviews, registration number: CRD42019134278). Findings will be shared via PROSPERO and a peer-reviewed journal.

## Results

The search terms have been piloted and finalized. After selecting and assessing the quality of the publications, data extraction and analyses will begin. Data synthesis and presentation of the findings will be completed by the end of 2020.

## Discussion

There is a high rate of prescription of testosterone despite its unclear effect on cardiovascular events in men. A thorough evaluation of the published trials, as guided by this protocol, will provide evidence-based recommendations to ascertain the real benefits, neutral effects, or adverse effects on the cardiovascular health of men. It is anticipated that the findings will facilitate effective and safe clinical management of hypogonadal men.

## Abbreviations

CINAHL: Cumulated Index to Nursing and Allied Health Literature
PRISMA: Preferred Reporting Items for Systematic Reviews and Meta-Analyses
PROSPERO: Prospective Register of Systematic Reviews
